# Global Assessment of the Activity of Tigecycline against Multidrug-Resistant Gram-Negative Pathogens between 2004 and 2014 as Part of the Tigecycline Evaluation and Surveillance Trial

**DOI:** 10.1128/mSphere.00310-16

**Published:** 2017-01-18

**Authors:** Anna Giammanco, Cinzia Calà, Teresa Fasciana, Michael J. Dowzicky

**Affiliations:** a Department of Sciences for Health Promotion and Mother and Child Care “G. D'Alessandro,” University of Palermo, Palermo, Italy; b Pfizer Inc., Collegeville, Pennsylvania, USA; Antimicrobial Development Specialists, LLC

**Keywords:** Gram-negative bacteria, multidrug resistance, surveillance studies, tigecycline

## Abstract

Multidrug-resistant (MDR) Gram-negative organisms are a burden on the global health care system. The Tigecycline Evaluation and Surveillance Trial (TEST) is an ongoing global study designed to monitor the *in vitro* activities of tigecycline and a panel of marketed antimicrobials against a range of clinically significant pathogens. In this study, *in vitro* data are presented for MDR *Acinetobacter baumannii*, *Pseudomonas aeruginosa*, *Escherichia coli*, *Klebsiella pneumoniae*, *Klebsiella oxytoca*, *Enterobacter aerogenes*, and *Enterobacter cloacae* isolates collected from 2004 to 2014. In total, 13% (21,967/170,759) of isolates displayed multidrug resistance globally, with the highest rates recorded among *A. baumannii* (overall rate, 44% [8,294/18,741], increasing from 23% [309/1,323] in 2004 to 63% [447/712] in 2014). Other multidrug resistance rates ranged from 2.5% for *K. oxytoca* (203/8,000) to 12% for *P. aeruginosa* and *K. pneumoniae* (3,951/32,786 and 3,895/32,888, respectively), and rates among these pathogens remained stable during the study period. Against MDR *E. coli*, *Klebsiella* spp., and *E. aerogenes*, the lowest rates of resistance were to tigecycline (0.2%, 6%, and 12%, respectively), and the lowest MIC_90_ value against *A. baumannii* was observed for tigecycline (2 mg/liter; MIC range, ≤0.008 to ≥32 mg/liter). The only significant change in resistance to tigecycline during the study period was for MDR *E. coli* (*P* < 0.01), among which eight resistant isolates were identified globally from 2009 to 2013. In summary, these results show that tigecycline retained *in vitro* activity against the majority of MDR Gram-negative organisms presented here, but the rising rates of MDR *A. baumannii* highlight the need for the continued monitoring of global multidrug resistance.

**IMPORTANCE** Multidrug resistance among bacterial pathogens is an ongoing global problem and renders antimicrobial agents ineffective at treating bacterial infections. In the health care setting, infections caused by multidrug-resistant (MDR) Gram-negative bacteria can cause increased mortality, longer hospital stays, and higher treatments costs. The aim of the Tigecycline Evaluation and Surveillance Trial (TEST) is to assess the *in vitro* antimicrobial activities of tigecycline and other contemporary agents against clinically relevant pathogens. This paper presents antimicrobial activity data from the TEST study between 2004 and 2014 and examines global rates of MDR Gram-negative isolates, including *Acinetobacter baumannii*, *Pseudomonas aeruginosa*, and members of the *Enterobacteriaceae*, during this time. Our results show that tigecycline retained *in vitro* activity against many MDR Gram-negative pathogens over the study period, while rates of MDR *A. baumannii* increased globally. Using these findings, we hope to highlight the current status of multidrug resistance in medical facilities worldwide.

## Abstract (precis)

Multidrug resistance among bacterial pathogens is an ongoing global problem and renders antimicrobial agents ineffective at treating bacterial infections. In the health care setting, infections caused by multidrug-resistant (MDR) Gram-negative bacteria can cause increased mortality, longer hospital stays, and higher treatments costs. The aim of the Tigecycline Evaluation and Surveillance Trial (TEST) is to assess the *in vitro* antimicrobial activities of tigecycline and other contemporary agents against clinically relevant pathogens. This paper presents antimicrobial activity data from the TEST study between 2004 and 2014 and examines global rates of MDR Gram-negative isolates, including *Acinetobacter baumannii*, *Pseudomonas aeruginosa*, and members of the *Enterobacteriaceae*, during this time. Our results show that tigecycline retained *in vitro* activity against many MDR Gram-negative pathogens over the study period, while rates of MDR *A. baumannii* increased globally. Using these findings, we hope to highlight the current status of multidrug resistance in medical facilities worldwide.

## INTRODUCTION

Multidrug resistance among Gram-negative organisms is a global problem, with rates of infections caused by multidrug-resistant (MDR) Gram-negative bacteria increasing worldwide (1–3). MDR Gram-negative pathogens, such as *Acinetobacter baumannii*, *Pseudomonas aeruginosa*, and the *Enterobacteriaceae*, are associated with increased lengths of hospitalization, higher health care costs, and greater rates of mortality (2, 4–6). These organisms have been highlighted as clinically important bacteria, and some are included among the ESKAPE pathogens (an acronym for *Enterococcus faecium*, *Staphylococcus aureus*, *Klebsiella pneumoniae*, *A. baumanii*, *Pseudomonas aeruginosa*, and *Enterobacter* spp.) (7).

Tigecycline is a broad-spectrum glycylcycline antimicrobial agent with *in vitro* activity against both Gram-positive and Gram-negative organisms. Tigecycline has been approved in the United States and Europe for the treatment of complicated skin and intra-abdominal infections and also in the United States for community-acquired bacterial pneumonia (8, 9). The *in vitro* activity of tigecycline is monitored globally, alongside comparator agents, against clinical Gram-positive and Gram-negative isolates as part of the Tigecycline Evaluation and Surveillance Trial (TEST). This study describes the activity of tigecycline against MDR Gram-negative isolates collected globally between 2004 and 2014. Isolates collected during the earlier years of the study period have been included in previous TEST publications, including reports focused on MDR *A. baumannii* (10) and MDR *Enterobacteriaceae* (11) isolates that were collected in the United States between 2004 and 2006 and on MDR Gram-negative isolates collected globally between 2004 and 2013 (12).

## RESULTS

Between 2004 and 2014, the majority of TEST centers were located in North America and Europe (37% and 36%, respectively). Over the study period, 13% (21,967/170,759) of Gram-negative isolates collected globally were MDR.

### *Acinetobacter baumannii*.

**FIG 1:**
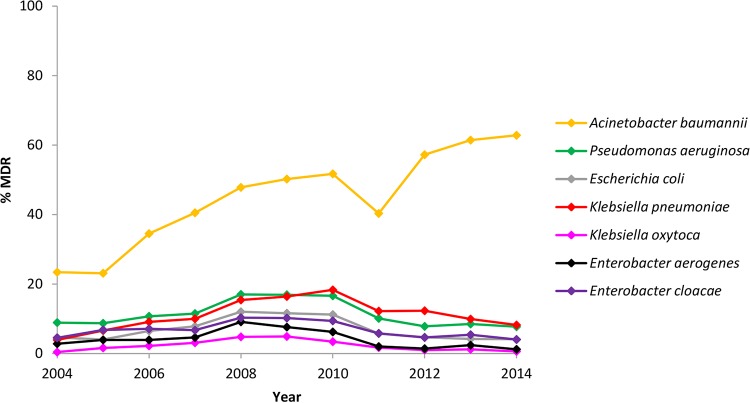
Changes in global rates of MDR Gram-negative isolates collected between 2004 and 2014.

In total, 18,741 isolates of *A. baumannii* were collected globally, of which 44% were reported to be MDR (Table 1). Global rates of MDR *A. baumannii* isolates increased during the study period, from 23% in 2004 to 63% in 2014 (Fig. 1). By region, overall multidrug resistance among *A. baumannii* was lowest in North America (31%) (Table 1). More than 50% of *A. baumannii* isolates collected in Africa, the Middle East, and Latin America were MDR.

**TABLE 1:** Regional and global rates of MDR Gram-negative isolates collected between 2004 and 2014

Organism and region	No. of centers^a^	No. of MDR isolates	Total no. of isolates	% MDR
*Acinetobacter baumannii*				
Africa	17	249	407	61.2
Asia-Pacific Rim	43	596	1,229	48.5
Europe	190	3,617	8,409	43.0
Latin America	58	1,560	2,213	70.5
Middle East	20	499	718	69.5
North America	169	1,773	5,765	30.8
Global	497	8,294	18,741	44.3
*Pseudomonas aeruginosa*				
Africa	16	73	558	13.1
Asia-Pacific Rim	46	327	1,772	18.5
Europe	192	1,777	14,951	11.9
Latin America	66	913	3,340	27.3
Middle East	21	129	989	13.0
North America	180	732	11,176	6.5
Global	521	3,951	32,786	12.1
*Escherichia coli*				
Africa	13	58	731	7.9
Asia-Pacific Rim	41	299	2,178	13.7
Europe	190	1,323	19,242	6.9
Latin America	65	795	4,492	17.7
Middle East	23	190	1,301	14.6
North America	157	557	14,317	3.9
Global	489	3,222	42,261	7.6
*Klebsiella pneumoniae*				
Africa	16	111	668	16.6
Asia-Pacific Rim	43	299	1,940	15.4
Europe	175	1,778	13,936	12.8
Latin America	62	709	3,704	19.1
Middle East	21	231	1,142	20.2
North America	145	767	11,498	6.7
Global	462	3,895	32,888	11.8
*Klebsiella oxytoca*				
Africa	2	2	85	2.4
Asia-Pacific Rim	8	14	245	5.7
Europe	62	113	4,639	2.4
Latin America	20	29	395	7.3
Middle East	8	14	106	13.2
North America	22	31	2,530	1.2
Global	122	203	8,000	2.5
*Enterobacter aerogenes*				
Africa	3	5	94	5.3
Asia-Pacific Rim	17	25	490	5.1
Europe	72	193	3,733	5.2
Latin America	25	72	583	12.3
Middle East	8	13	256	5.1
North America	48	73	3,297	2.2
Global	173	381	8,453	4.5
*Enterobacter cloacae*				
Africa	11	37	494	7.5
Asia-Pacific Rim	36	122	1,437	8.5
Europe	168	978	13,205	7.4
Latin America	55	387	2,771	14.0
Middle East	15	49	815	6.0
North America	143	448	8,908	5.0
Global	428	2,021	27,630	7.3

a The number of TEST centers submitting MDR isolates. Not all centers submitted isolates during all study years. The Asia-Pacific Rim centers did not participate in TEST after 2010.

Overall, 95% of MDR *A. baumannii* isolates were resistant to ceftriaxone, and approximately 90% of isolates were resistant to ceftazidime, levofloxacin, meropenem, and piperacillin-tazobactam (Table 2). Global resistance to levofloxacin increased significantly from 92% (283/309) in 2004 to 96% (430/447) in 2014, and resistance to piperacillin-tazobactam increased significantly from 82% (252/309) in 2004 to 94% (422/447) in 2014 (*P* < 0.0001) (Table 3). The lowest levels of global resistance were reported for minocycline (13%). The lowest MIC_90_ value was observed for tigecycline (2 mg/liter), for which no breakpoints are available against *A. baumannii*.

**TABLE 2:** Global antimicrobial activity against MDR Gram-negative isolates collected between 2004 and 2014

Organism (no. of isolates) and antimicrobial agent	MIC (mg/liter) data		Susceptibility^a^		
	MIC_90_	Range	% S	% I	% R
*Acinetobacter baumannii* (8,294)					
Amikacin	≥128	≤0.5 to ≥128	20.1	7.7	72.2
Amoxicillin-clavulanic acid	≥64	1 to ≥64	—^b^	—	—
Ampicillin	≥64	≤0.5 to ≥64	—	—	—
Cefepime	≥64	≤0.5 to ≥64	5.3	13.6	81.2
Ceftazidime	≥64	≤1 to ≥64	4.1	5.3	90.7
Ceftriaxone	≥128	≤0.06 to ≥128	0.7	4.8	94.6
Levofloxacin	≥16	0.03 to ≥16	2.3	7.9	89.8
Meropenem (7,338)^c^	≥32	≤0.06 to ≥32	6.9	3.0	90.1
Minocycline	16	≤0.5 to ≥32	68.6	18.9	12.6
Piperacillin-tazobactam	≥256	≤0.06 to ≥256	2.7	6.4	90.9
Tigecycline	2	≤0.008 to ≥32	—	—	—
*Pseudomonas aeruginosa* (3,951)					
Amikacin	≥128	≤0.5 to ≥128	46.1	10.2	43.7
Amoxicillin-clavulanic acid	≥64	1 to ≥64	—	—	—
Ampicillin	≥64	≤0.5 to ≥64	—	—	—
Cefepime	≥64	≤0.5 to ≥64	7.7	22.5	69.8
Ceftazidime	≥64	≤1 to ≥64	11.5	11.0	77.5
Ceftriaxone	≥128	≤0.06 to ≥128	—	—	—
Levofloxacin	≥16	0.03 to ≥16	2.1	1.5	96.4
Meropenem (3,392)^c^	≥32	≤0.06 to ≥32	4.5	2.9	92.6
Minocycline	≥32	≤0.5 to ≥32	—	—	—
Piperacillin-tazobactam	≥256	0.25 to ≥256	10.8	22.3	66.9
Tigecycline	≥32	≤0.008 to ≥32	—	—	—
*Escherichia coli* (3,222)					
Amikacin	32	≤0.5 to ≥128	88.3	2.7	9.0
Amoxicillin-clavulanic acid	≥64	1 to ≥64	22.7	36.8	40.4
Ampicillin	≥64	≤0.5 to ≥64	0.6	0.1	99.3
Cefepime	≥64	≤0.5 to ≥64	49.6	12.6	37.8
Ceftriaxone	≥128	≤0.06 to ≥128	41.5	1.6	57.0
Levofloxacin	≥16	≤0.008 to ≥16	1.4	0.2	98.4
Meropenem (2,814)^c^	0.25	≤0.06 to ≥32	92.9	1.1	6.0
Minocycline	≥32	≤0.5 to ≥32	5.2	2.0	92.8
Piperacillin-tazobactam	≥256	0.25 to ≥256	68.2	14.1	17.7
Tigecycline	1	≤0.008 to ≥32	99.5	0.2	0.2
*Klebsiella pneumoniae* (3,895)					
Amikacin	≥128	≤0.5 to ≥128	68.5	11.8	19.7
Amoxicillin-clavulanic acid	≥64	0.5 to ≥64	10.7	19.5	69.8
Ampicillin	≥64	2 to ≥64	0.0	0.1	99.9
Cefepime	≥64	≤0.5 to ≥64	15.0	10.2	74.8
Ceftriaxone	≥128	≤0.06 to ≥128	10.1	0.9	89.0
Levofloxacin	≥16	0.03 to ≥16	4.4	1.3	94.3
Meropenem (3,578)^c^	≥32	≤0.06 to ≥32	57.0	3.5	39.5
Minocycline	≥32	≤0.5 to ≥32	25.5	9.8	64.7
Piperacillin-tazobactam	≥256	0.12 to ≥256	21.5	13.7	64.8
Tigecycline	4	≤0.008 to ≥32	83.0	11.3	5.7
*Klebsiella oxytoca* (203)					
Amikacin	≥128	≤0.5 to ≥128	76.8	5.4	17.7
Amoxicillin-clavulanic acid	≥64	0.25 to ≥64	12.8	21.7	65.5
Ampicillin	≥64	32 to ≥64	0.0	0.0	100
Cefepime	≥64	≤0.5 to ≥64	25.6	28.1	46.3
Ceftriaxone	≥128	≤0.06 to ≥128	13.3	0.0	86.7
Levofloxacin	≥16	0.06 to ≥16	5.4	3.4	91.1
Meropenem (180)^c^	8	≤0.06 to ≥32	80.0	2.2	17.8
Minocycline	≥32	≤0.5 to ≥32	7.4	6.9	85.7
Piperacillin-tazobactam	≥256	≤0.06 to ≥256	34.5	12.8	52.7
Tigecycline	4	0.12 to 8	81.3	12.8	5.9
*Enterobacter aerogenes* (381)					
Amikacin	≥128	≤0.5 to ≥128	74.0	5.0	21.0
Amoxicillin-clavulanic acid	≥64	2 to ≥64	2.1	4.5	93.4
Ampicillin	≥64	16 to ≥64	0.0	0.5	99.5
Cefepime	≥64	≤0.5 to ≥64	38.3	21.3	40.4
Ceftriaxone	≥128	≤0.06 to ≥128	12.3	0.8	86.9
Levofloxacin	≥16	0.06 to ≥16	13.9	2.9	83.2
Meropenem (321)^c^	16	≤0.06 to ≥32	67.9	3.1	29.0
Minocycline	≥32	≤0.5 to ≥32	15.0	8.1	76.9
Piperacillin-tazobactam	≥256	1 to ≥256	28.6	30.2	41.2
Tigecycline	8	0.015 to 16	66.9	20.7	12.3
*Enterobacter cloacae* (2,021)					
Amikacin	≥128	≤0.5 to ≥128	75.4	4.4	20.2
Amoxicillin-clavulanic acid	≥64	0.25 to ≥64	0.5	1.9	97.6
Ampicillin	≥64	≤0.5 to ≥64	0.4	0.5	99.0
Cefepime	≥64	≤0.5 to ≥64	23.5	30.8	45.7
Ceftriaxone	≥128	≤0.06 to ≥128	7.7	1.9	90.5
Levofloxacin	≥16	≤0.008 to ≥16	11.2	3.4	85.4
Meropenem (1,717)^c^	8	≤0.06 to ≥32	79.1	3.8	17.1
Minocycline	≥32	≤0.5 to ≥32	5.3	6.0	88.6
Piperacillin-tazobactam	≥256	≤0.06 to ≥256	26.0	20.8	53.2
Tigecycline	8	0.015 to ≥32	64.4	20.3	15.3

a S, susceptible; I, intermediate susceptibility; R, resistant.

b —, no breakpoints available.

c Susceptibility data for imipenem were collected from 2004 to 2006, after which time imipenem was replaced with meropenem.

**TABLE 3:** Statistically significant changes in global antimicrobial activity among MDR Gram-negative isolates collected between 2004 and 2014, by study year

Species and antimicrobial agent^a^	% of isolates resistant to the indicated drug in:											*P* value^b^	Change in resistance
	2004	2005	2006	2007	2008	2009	2010	2011	2012	2013	2014		
*A. baumannii*	309	310	645	987	1,134	1,379	1,112	588	661	722	447		
Cefepime	79.0	83.9	82.0	85.4	72.4	72.2	81.3	85.5	89.0	90.3	87.7	<0.0001	Increased
Levofloxacin	91.6	92.6	83.9	87.1	88.4	89.2	88.0	91.7	93.2	94.5	96.2	<0.0001	Increased
Meropenem^d^	*—^c^* (2)	89.5 (19)	87.6 (307)	86.8 (967)	85.4	85.1	91.6	95.1	95.3	96.8	97.5	<0.0001	Increased
Minocycline	10.4	8.4	9.8	11.3	11.3	10.9	17.0	13.3	13.8	15.2	13.9	<0.0001	Increased
Pip-taz	81.6	66.1	80.9	87.1	93.1	93.0	95.8	94.7	94.6	96.3	94.4	<0.0001	Increased
													
*P. aeruginosa*	10	7	147	464	656	726	541	256	189	251	145		
Meropenem^d^	—	—	95.9	91.2	90.2	91.5	90.4	96.5	97.4	96.4	98.6	<0.0001	Increased
													
*E. coli*	117	116	268	403	578	651	479	195	154	162	99		
Amikacin	4.3	10.3	8.6	14.1	13.7	10.0	4.8	2.1	1.9	7.4	6.1	<0.0001	Decreased
Cefepime	15.4	27.6	34.3	38.7	39.8	38.2	37.4	40.5	45.5	41.4	46.5	<0.0001	Increased
Tigecycline	0.0	0.0	0.0	0.0	0.0	0.2	0.2	1.0	1.3	1.2	0.0	<0.01	Increased
													
*K. pneumoniae*	83	151	281	410	599	717	614	306	307	280	147		
Amikacin	14.5	25.8	26.0	28.0	29.5	24.1	10.1	9.5	9.4	16.4	8.2	<0.0001	Decreased
Amoxy-clav	55.4	66.9	65.5	68.5	67.1	67.2	70.7	73.9	77.2	76.1	76.9	<0.0001	Increased
Cefepime	61.4	69.5	71.2	74.6	73.6	71.0	72.1	76.8	82.7	85.0	88.4	<0.0001	Increased
Levofloxacin	85.5	90.7	91.1	93.7	93.5	94.1	97.7	94.8	95.4	95.0	96.6	<0.0001	Increased
Meropenem^d^	41.2 (17)	51.7 (29)	45.2 (155)	36.1 (407)	26.4	30.3	28.2	52.6	62.5	65.0	63.3	<0.0001	Increased
Minocycline	68.7	53.0	60.9	65.1	77.0	73.9	78.5	54.2	41.4	42.9	40.8	<0.0001	Decreased
Pip-taz	50.6	55.6	64.8	65.9	63.1	59.1	61.6	69.0	75.9	75.7	74.8	<0.0001	Increased
													
*K. oxytoca*	2	8	16	30	39	50	25	13	7	10	3		
Amikacin	—	—	43.8	30.0	25.6	10.0	8.0	0.0	—	—	—	<0.001	Decreased
													
*E. cloacae*	78	124	181	234	339	379	280	131	90	128	57		
Cefepime	35.9	29.8	48.1	48.3	49.9	43.0	42.5	41.2	47.8	57.8	63.2	<0.01	Increased

a For each bacterial species, data in the shaded rows indicate the species and the number of isolates collected (by year). Abbreviations for antimicrobial agents: Pip-taz, piperacillin-tazobactam; Amoxy-clav, amoxicillin-clavulanic acid.

b A cutoff value of *P* < 0.01 was used for statistical significance testing.

c —, the percent resistance was not calculated when ≤10 isolates of the species were collected that year.

d Susceptibility data for imipenem were collected from 2004 to 2006, after which time imipenem was replaced with meropenem. The values in parentheses indicate the numbers of isolates tested against meropenem.

### *Pseudomonas aeruginosa*.

MDR isolates accounted for 12% of the 32,786 *P. aeruginosa* total isolates submitted (Table 1). Global rates of MDR *P. aeruginosa* increased from 9% in the 2004-2005 period to 17% in the 2008-2010 period, and then the rate decreased to 8% in 2014 (Fig. 1). Regionally, overall rates of multidrug resistance among *P. aeruginosa* were lowest in North America (7%) and highest in Latin America (27%) (Table 1). Among MDR *P. aeruginosa* isolates, the highest levels of global resistance were reported to meropenem and levofloxacin (92% to 96%) (Table 2). All agents had limited activity against isolates of MDR* P. aeruginosa* (MIC_90_, ≥16 mg/liter).

### *Escherichia coli*.

Participating centers submitted a total of 42,261 *E. coli* isolates, of which 8% were MDR (Table 1). Globally, rates of MDR *E. coli* increased from 5% in 2004 to 12% in the 2008-2009 period, and then decreased to 4% in the 2013-2014 period (Fig. 1). Regional percentages of MDR *E. coli* ranged from 4% in North America to 18% in Latin America (Table 1). Globally, nearly all MDR *E. coli* isolates tested were resistant to levofloxacin and ampicillin (≥98%), and 93% of isolates were resistant to minocycline (Table 2). Global resistance to cefepime increased significantly from 15% (18/117) in 2004 to 46% (46/99) in 2014 (*P* < 0.0001) (Table 3). The lowest level of resistance globally was to tigecycline (0.2%). No tigecycline-resistant isolates were identified between 2004 and 2008; however, eight resistant isolates were identified across regions between 2009 and 2013 (Africa in 2012 [*n* = 1], Europe in 2010 [*n* = 1], Latin America in 2009 [*n* = 1], the Middle East in 2011 [*n* = 1], and North America in 2011 [*n* = 1], 2012 [*n* = 1], and 2013 [*n* = 2]). This change was statistically significant (*P* < 0.01) (Table 3).

### *Klebsiella pneumoniae*.

Multidrug resistance was reported in 12% of 32,888 of *K. pneumoniae* isolates submitted globally (Table 1). During the study period, global rates of MDR *K. pneumoniae* increased from 4% in 2004 to 18% in 2010, and then the rate decreased to 8% in 2014 (Fig. 1). By region, the lowest rates of MDR *K. pneumoniae* were found in North America (7%), with the highest rates in Latin America and the Middle East (19% and 20%, respectively) (Table 1). High levels of global resistance were reported to ceftriaxone and levofloxacin (89% and 94%, respectively) (Table 2). Global resistance to amoxicillin-clavulanic acid, cefepime, levofloxacin, and piperacillin-tazobactam increased significantly during the study period: amoxicillin-clavulanic acid, 55% (46/83) in 2004 to 77% (113/147) in 2014; cefepime, 61% (51/83) in 2004 to 88% (130/147) in 2014; levofloxacin, 86% (71/83) in 2004 to 97% (142/147) in 2014; piperacillin-tazobactam, 51% (42/83) in 2004 to 75% (110/147) in 2014 (*P* < 0.0001) (Table 3). The lowest rate of resistance among MDR* K. pneumoniae* was to tigecycline (6%).

### *Klebsiella oxytoca*.

Over the study period, 8,000 isolates of *K. oxytoca* were submitted globally, of which 2.5% were MDR (Table 1). The global rates of MDR *K. oxytoca* increased from 0.4% in 2004 to 5% in the 2008-2009 period and then decreased to 0.6% in 2014 (Fig. 1). The highest percentages of MDR *K. oxytoca* isolates were reported in the Middle East (13%), while rates in all other regions were ≤7% (Table 1). More than 80% of MDR *K. oxytoca* isolates collected globally were resistant to minocycline, ceftriaxone, and levofloxacin (86% to 91%) (Table 2). The lowest rate of resistance was to tigecycline (6%).

### *Enterobacter aerogenes*.

Overall, 4.5% of 8,453 *E. aerogenes* isolates collected from all regions were MDR (Table 1). Globally, the rates of MDR *E. aerogenes* increased from 3% in 2004 to 9% in 2008 but then decreased to 1% in 2014 (Fig. 1). There was a rate of 12% MDR *E. aerogenes* in Latin America and a rate of ≤5% in all other regions (Table 1). More than 80% of isolates were resistant to levofloxacin and ceftriaxone (83% and 87%, respectively) (Table 2). The lowest levels of resistance were reported to tigecycline (12%), followed by amikacin (21%).

### *Enterobacter cloacae*.

Among the 27,630 isolates of *E. cloacae* submitted globally, a total of 7% were MDR (Table 1). Global rates of MDR *E. cloacae* increased from 4.5% in 2004 to 10% in the 2008-2009 period, and then decreased to 4% in 2014 (Fig. 1). In Latin America, 14% of *E. cloacae* isolates were MDR, compared with <9% of isolates in all other regions (Table 1). The majority of MDR *E. cloacae* isolates collected globally were resistant to levofloxacin (85%), minocycline (89%), and ceftriaxone (90%) (Table 2). Global resistance to cefepime increased significantly, from 36% (28/78) in 2004 to 63% (36/57) in 2014 (*P* < 0.01) (Table 3). Resistance to tigecycline was the lowest reported rate (15%).

## DISCUSSION

This study describes the global rates of multidrug resistance among a selection of clinically important Gram-negative organisms collected between 2004 and 2014, and it shows the *in vitro* antimicrobial activity of tigecycline and a panel of other contemporary antimicrobial agents against these resistant isolates.

Tigecycline retained *in vitro* activity against the majority of MDR organisms collected between 2004 and 2014, with the exception of *P. aeruginosa* (MIC_90_, ≥32 mg/liter), against which tigecycline is known to have limited activity (13). Furthermore, none of the antimicrobial agents tested in the present study demonstrated potent *in vitro* activity against MDR *P. aeruginosa*, which was highlighted among the ESKAPE organisms as a cause for global concern (7). Against MDR *A. baumannii*, another of the ESKAPE pathogens, tigecycline had the lowest MIC_90_ (2 mg/liter) of the agents on the TEST panel. This MIC_90_ was comparable with that reported from a global study of *A. baumannii* isolates collected between 2005 and 2009, which showed that tigecycline inhibited 95% of MDR *Acinetobacter* spp. isolates at ≤2 mg/liter (14). Also, a study by Mammina et al. (15) of MDR *A. baumannii* from Palermo, Italy, reported tigecycline MICs between ≤0.5 mg/liter and 4 mg/liter.

Among the MDR *Enterobacteriaceae* collected in this study, rates of resistance were lowest to tigecycline. The overall global rates of tigecycline resistance among MDR *Enterobacteriaceae* were 15% (357/2,402) for *Enterobacter* spp., 6% (235/4,098) for *Klebsiella* spp., and 0.2% (8/3,222) for *E. coli*. Although the highest rates of tigecycline resistance among MDR *Enterobacteriaceae* were recorded for *Enterobacter* spp., the yearly rates of resistance among *E. aerogenes* and *E. cloacae* isolates fluctuated between 1% and 42% during the study period, and this could be explained by the low numbers of isolates submitted in some years. By year, the number of MDR *E. aerogenes* isolates collected ranged from 6 in 2014 to 83 in 2008. Yearly totals of MDR *E. cloacae* isolates were higher than those for MDR *E. aerogenes* isolates, but these totals only exceeded 200 isolates in four out of seven study years (2007 to 2010).

The overall global rate of tigecycline resistance among isolates of MDR *Klebsiella* spp. in the current study was 6% (*K. oxytoca*, 12/203; *K. pneumoniae*, 223/3,895). For MDR *K. oxytoca* isolates, the overall rate of tigecycline resistance may be difficult to interpret due to low isolate numbers (≤50 isolates collected per study year). Furthermore, tigecycline-resistant isolates were only identified in 2006 (19% [3/16]), 2008 (10% [4/39]), 2009 (4% [2/50]), and 2011 (23% [3/13]). Two of the three tigecycline-resistant *K. oxytoca* isolates collected in North America and Europe in 2006 and 2008, respectively, were submitted by the same center from each region. This suggests a localized incidence of tigecycline resistance during these two study years.

Higher numbers of MDR *K. pneumoniae* isolates were collected than numbers of MDR *K. oxytoca* isolates, and global rates of tigecycline-resistant MDR *K. pneumoniae* isolates ranged from 3% (4/147) to 11% (9/83). Despite this, the rates of tigecycline-resistant MDR *K. pneumoniae* isolates decreased from 9% (27/307) in 2012 to 3% (4/147) in 2014, which may signify the start of a decline in global resistance. Further surveillance will be needed to follow this trend. In their study of *bla*_KPC_-carrying *K. pneumoniae* in Palermo, Italy, Bonura et al. (16) reported a shift toward a polyclonal epidemic, which highlights that the evolution of resistance is complex and that the importance of changing patterns of resistance should not be underestimated.

In this study, the identification of tigecycline-resistant MDR *E. coli* from 2009 onwards indicates that these organisms have recently acquired mechanisms of resistance to the glycylcyclines. In the literature, occurrences of emerging tigecycline resistance among patients with *E. coli* infections have been reported in the United Kingdom (17) and in Italy (18). In both cases, *E. coli* isolates that were initially susceptible to tigecycline *in vivo* became tigecycline resistant after prolonged antimicrobial administration and, furthermore, *in vitro* the resistant isolates were found to produce carbapenemases: New Delhi metallo-β-lactamase 1 (NDM-1) (17) and *K. pneumoniae* carbapenemase 3 (KPC-3) (18). These carbapenemases are active against third-generation cephalosporins and carbapenems; therefore, the acquisition of tigecycline resistance likely confers an MDR phenotype. Stone et al. (17) reported the development of resistance *in vivo* after 53 days of tigecycline treatment, and Spanu et al. (18) reported resistance after 21 days of treatment. Despite these reports of development of tigecycline resistance among *E. coli* isolates, the current TEST study shows that tigecycline remains active against the majority of MDR *E. coli* isolates, and the TEST publication by Hoban et al. (12) reported that tigecycline was active against carbapenem-resistant *E. coli* isolates collected between 2004 and 2013.

By organism, the highest overall rates of multidrug resistance reported in the present study were among *A. baumannii* isolates, for which 44% of isolates collected globally were MDR. By year, the results presented in Fig. 1 show an increase in the rates of MDR *A. baumannii* during the study period, from 23% (309/1,323) in 2004 to 63% (447/712) in 2014. The previous TEST publication by Garrison et al. (19) reported increasing global rates of MDR *A. baumannii* isolates between 2004 and 2007, and Mendes et al. (14) described a global increase in the rates of MDR *Acinetobacter* spp. between 2005 and 2009. Our report shows that multidrug resistance among *A. baumannii* isolates continues to increase; given the limited treatment options for infections caused by such organisms, this is a cause for concern.

The majority of MDR isolates collected globally were resistant to levofloxacin, with rates of resistance ranging from 83% of *E. aerogenes* isolates to 98% of *E. coli*. The World Health Organization (WHO) recently published a report on global antimicrobial resistance that included national data on rates of resistance from Africa, the Americas, Eastern Mediterranean, Europe, Southeast Asia, and the Western Pacific (20). In their report, resistance rates among *E. coli* isolates of greater than 50% were reported to fluoroquinolones in all regions except Europe. They also showed that infections caused by fluoroquinolone-resistant *E. coli* isolates were associated with increased mortality. Fluoroquinolone resistance has been linked with extended-spectrum β-lactamase production among the *Enterobacteriaceae* (4, 6); therefore, extended-spectrum β-lactamase production may be an indicator of multidrug resistance.

Although global surveillance studies, such as TEST, have reported important information on changes in antimicrobial activity and resistance, there are certain limitations to the data presented. One such limitation is the yearly variation in the numbers of participating centers. For example, the Asia-Pacific region stopped submitting isolates between 2010 and 2014. In this region, the rates of MDR *A. baumannii* isolates increased from 29% (14/49) in 2005 to 60% (97/161) during the final year of participation. The lack of isolates from this region after 2010 will have impacted the global results. Furthermore, the majority of centers participating in TEST were located in Europe and North America; therefore, changes in these regions could have had a greater impact on the global data.

Despite these limitations, the data presented in this study show that tigecycline has remained active against this global collection of Gram-negative pathogens. The collection of small numbers of tigecycline-resistant MDR *E. coli* isolates is of concern, however, and highlights the importance for the continued surveillance of tigecycline activity globally. The increasing rates of MDR *A. baumannii* isolates must also be monitored, and this information should be used to aid health care facilities in reducing MDR infections worldwide. Overall, more global studies of MDR pathogens are needed if the ongoing problem of antimicrobial resistance is to be addressed.

## MATERIALS AND METHODS

A total of 611 TEST centers submitted MDR Gram-negative isolates between 2004 and 2014. The numbers of centers located in each study region were as follows: Africa, 20; Asia-Pacific Rim, 52; Europe, 219; Latin America, 69; the Middle East, 23; North America, 228. Not all study centers submitted isolates during all study years. Centers from the Asia-Pacific Rim did not participate in the study after 2010. Isolates were collected from all body sites from patients with known hospital- or community-acquired infections.

MICs were determined in local laboratories using Clinical and Laboratory Standards Institute (CLSI) guidelines for the broth microdilution methodology (21). Antimicrobial susceptibility was assessed using breakpoints approved by the CLSI (22), except for tigecycline, for which the U.S. Food and Drug Administration (FDA) breakpoints were used (8). Breakpoints were not available for tigecycline against *A. baumannii* or *P. aeruginosa* isolates. Full methodology details for the TEST study have been published previously (23).

In the current study, multidrug resistance was defined as resistance to three or more classes of antimicrobial agents. The classes used to define MDR isolates among the *Enterobacteriaceae* were aminoglycosides (amikacin), β-lactams (ampicillin, amoxicillin-clavulanic acid, cefepime, ceftriaxone, or piperacillin-tazobactam), carbapenems (imipenem/meropenem), fluoroquinolones (levofloxacin), glycylcyclines (tigecycline), and tetracyclines (minocycline); the classes used to define MDR *A. baumannii* isolates were aminoglycosides (amikacin), β-lactams (cefepime, ceftazidime, ceftriaxone, or piperacillin-tazobactam), carbapenems (imipenem/meropenem), fluoroquinolones (levofloxacin), and tetracyclines (minocycline); the classes used to define MDR *P. aeruginosa* isolates were aminoglycosides (amikacin), β-lactams (cefepime, ceftazidime, or piperacillin-tazobactam), carbapenems (imipenem/meropenem), and fluoroquinolones (levofloxacin).

The Cochran-Armitage trend test was used to identify statistically significant changes in susceptibility between 2004 and 2014, with a cutoff value of *P* < 0.01 to indicate significance, due to the large number of trend tests performed.
